# Platelets disrupt vasculogenic mimicry by cancer cells

**DOI:** 10.1038/s41598-020-62648-x

**Published:** 2020-04-03

**Authors:** Carmela Martini, Emma J. Thompson, Stephanie R. Hyslop, Michaelia P. Cockshell, Brian J. Dale, Lisa M. Ebert, Anthony E. Woods, Emma C. Josefsson, Claudine S. Bonder

**Affiliations:** 1 Centre for Cancer Biology, University of South Australia and SA Pathology, Adelaide, SA Australia; 2 School of Pharmacy and Medical Sciences, University of South Australia, Adelaide, SA Australia; 3 The Walter and Eliza Hall Institute of Medical Research, Parkville, VIC Australia; 4 Department of Medical Biology, The University of Melbourne, Melbourne, VIC Australia; 5 Adelaide Medical School, University of Adelaide, Adelaide, SA Australia

**Keywords:** Metastasis, Tumour angiogenesis, Mechanisms of disease

## Abstract

Tumour vasculature supports the growth and progression of solid cancers with both angiogenesis (endothelial cell proliferation) and vasculogenic mimicry (VM, the formation of vascular structures by cancer cells themselves) predictors of poor patient outcomes. Increased circulating platelet counts also predict poor outcome for cancer patients but the influence of platelets on tumour vasculature is incompletely understood. Herein, we show with *in vitro* assays that platelets did not influence angiogenesis but did actively inhibit VM formation by cancer cell lines. Both platelet sized beads and the releasates from platelets were partially effective at inhibiting VM formation suggesting that direct contact maximises the effect. Platelets also promoted cancer cell invasion *in vitro*. B16F10 melanomas in *Bcl-x*^*Plt20/Plt20*^ thrombocytopenic mice showed a higher content of VM than their wildtype counterparts while angiogenesis did not differ. In a xenograft mouse model of breast cancer with low-dose aspirin to inactivate the platelets, the burden of MDA-MB-231-LM2 breast cancer cells was reduced and the gene expression profile of the cancer cells was altered; but no effect on tumour vasculature was observed. Taken together, this study provides new insights into the action of platelets on VM formation and their involvement in cancer progression.

## Introduction

Increased platelet count (thrombocytosis) is well documented to be associated with poor outcome for cancer patients^1–4^. Platelets promote tumourigenesis and metastasis via a number of complementary mechanisms, including (i) aggregation around the circulating cancer cells to form a platelet “cloak” thus shielding them from high shear forces generated by blood flow, lodging them into the vessel wall^5^ and protecting them from attack by the immune system^6^, (ii) the release of permeability factors and degradative enzymes that assist tumour cell extravasation from the circulation^7,8^, and (iii) the release of growth and angiogenic factors to facilitate the establishment of secondary tumours^9,10^. Platelet dysfunction and thrombotic disorders, such as thromboembolism, are recognized as important manifestations of cancer progression with platelet hyperactivity an early diagnostic feature of cancer and a major cause of death for these patients^11^. These and other observations of platelets supporting cancer cell survival and spreading, underpin the notion that platelets are important, if not essential, in the development of cancer metastasis. Clearly, understanding the molecular mechanisms by which platelets contribute to cancer progression is paramount to fighting this deadly group of diseases.

Cancer metastasis to distant organs relies on interactions between tumour cells and the host microenvironment. A particularly important relationship exists between cancer cells and the endothelial cell (EC) lined vasculature with angiogenesis being critical for cancer growth and metastasis^12,13^. As evidence of this, highly vascularised tumours predict poor outcomes for patients^12^. Activated platelets are a rich source of pro-angiogenic factors, including vascular endothelial growth factor A (VEGF-A), fibroblast growth factor 2 (FGF2) and platelet-derived growth factor (PDGF); but they also contain and release anti-angiogenic (angiostatic) molecules, such as thrombospondin (THBS1), plasminogen activator inhibitor 1 (PAI1) and endostatin^14^. These molecules, and many more, are stored in distinct α-granules in the platelets and can be selectively released^15^. Ho-Tin-Noe and colleagues demonstrated in mouse models of melanoma and lung cancer, that platelets not only promote angiogenesis but also actively prevent tumour haemorrhage by secreting angiopoietin-1 and serotonin^16^. A more recent study by Jiang and coworkers documented that platelet releasates increase breast cancer cell proliferation through VEGF-integrin signalling and enhance cancer cell-induced angiogenesis and tumour growth *in vivo*^17^. Notably, in tumours, platelet activation largely occurs at sites of vascular hyperpermeability where plasma leakage permits contact between collagen and the cancer cells^18^.

Aspirin, a widely used anti-platelet and non-steroidal anti-inflammatory drug (NSAID)^19^, has emerged as a promising drug for cancer prevention^20,21^. This drug has long been characterized as an irreversible cyclooxygenase (COX) inhibitor that reduces the synthesis of prostanoids, such as prostaglandin E2 and thromboxane A2, from arachidonic acid^22^. There is also building evidence that aspirin has COX-independent mechanisms of action in cancer cells causing changes in NFκB, RUNX1 and apoptosis^23–25^. These broad acting reactions are attributed to a dosing effect with 75 mg being anti-platelet, 325–600 mg being analgesic and 1.2 g being anti-inflammatory^26^. Etulain and colleagues reported that following thrombin activation of platelets, the ensuing platelet releasate promotes angiogenesis *in vitro*, and that this process can be inhibited by aspirin independently of VEGF^27^. In contrast, an earlier report from Abdelrahim and coworkers showed that other COX inhibitors (not aspirin) directly suppress VEGF expression by cancer cells to reduce angiogenesis^28^. Clearly, understanding the molecular mechanisms by which platelets and aspirin mediate tumour vascularisation is key to fighting cancer.

There is increasing evidence that tumour vasculature is comprised of not only of EC-lined vessels, but also non-EC lined vascular-like structures formed by cancer cells via a process called vasculogenic mimicry (VM)^29,30^. This process was initially identified in uveal melanoma^30^ with a recent meta-analysis of 5 year survival of >3600 patients across 11 different cancer types confirming VM in most solid tumours and correlating with poor prognosis^31^. Laser capture and gene expression profiling of VM-competent melanoma has identified genes associated with angiogenesis, stem cells, the extracellular matrix and hypoxia-related signalling pathways^32^, but a single VM defining biomarker is yet to be identified. VM networks are often contiguous with the EC-lined vasculature and both provide passage for erythrocytes and leukocytes as confirmed via Doppler imaging, intravital microscopy and magnetic resonance imaging^33–36^. The effect of platelets on VM in cancer remains to be determined.

In this study we use melanoma and breast cancer cell lines in *in vitro* assays to investigate the role of platelets in VM formation. We examine whether established VM can be influenced by the addition of platelets and whether platelet releasates are equally effective in modulating VM. We investigate VM formation *in vivo* by melanoma cells in mice with persistent thrombocytopenia. We also use the MDA-MB-231-LM2 cells in a xenograft model of breast cancer to monitor tumour growth, metastasis and the VM gene profile in mice treated without or with the platelet-inactivating aspirin.

## Results

### Involvement of platelets in angiogenesis and vasculogenic mimicry by cancer cells *in vitro*

**Figure 1:**
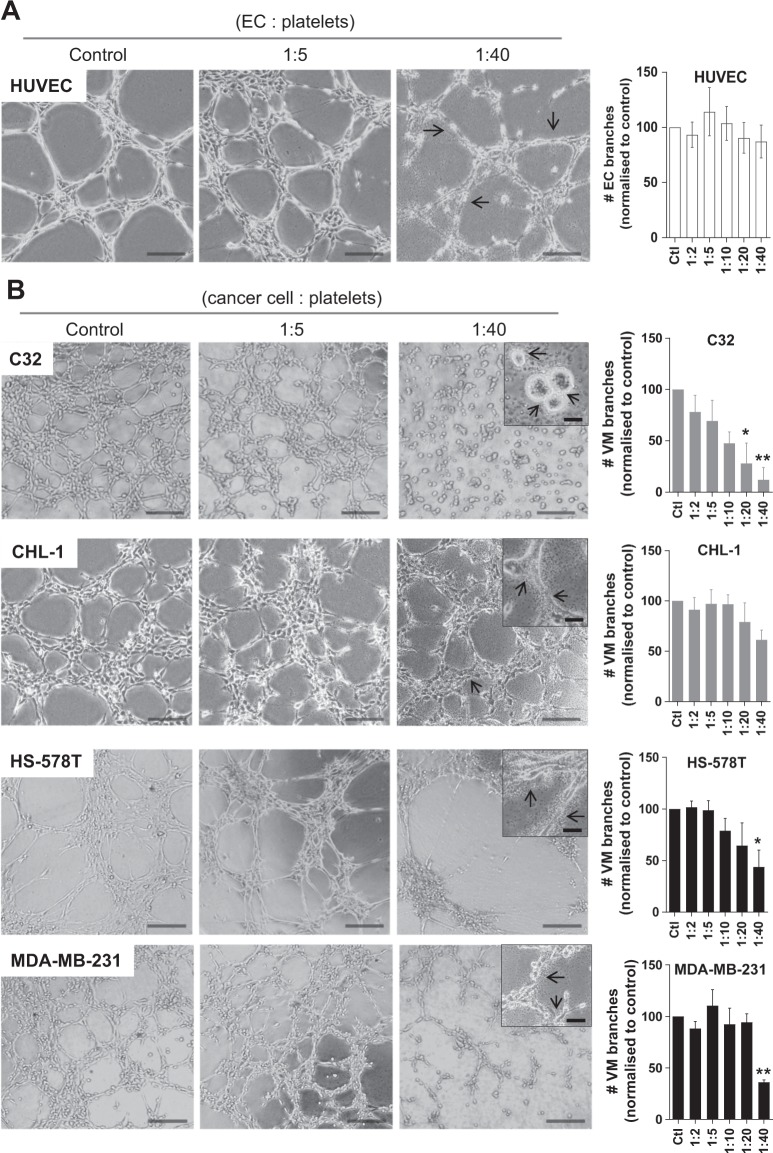
The influence of platelets on angiogenesis and vasculogenic mimcry *in vitro*. (**A**) Representative images of HUVECs co-cultured without or with platelets on Geltrex at the indicated ratio (EC:platelets). Arrows identify platelets in association with capillary-like structures and number of EC branches normalized to HUVEC only controls. (**B**) VM formation by melanoma C32 and CHL-1 cells as well as breast cancer HS-578T and MDA-MB-231 cells co-cultured without or with platelets (cells:platelets). Black arrows indicate platelet-cancer cell aggregates and platelet contact with tumour cells in VM structures. One-way ANOVA, data are expressed as mean ± SEM. Results are pooled from 3 separate experiments (different platelet donors). *p < 0.05, **p < 0.01, one-way ANOVA. Scale bar is 200 µm (40 µm for insert), original magnification 40x (400x for insert).

To appropriately investigate the effect of platelets on EC angiogenesis, we first confirmed that platelets isolated from the blood of healthy volunteers were ‘unstimulated’ as defined by a low-level surface expression of P-selectin (Supplementary Fig. 1). To avoid any influence by growth factors present within our angiogenesis assay, we utilised a growth factor-reduced formulation of Geltrex^TM^ (a Matrigel equivalent of secreted extracellular matrix proteins purified from murine Engelbreth-Holm-Swarm tumour cells^37^). As shown in Fig. 1A, unstimulated platelets co-cultured with human umbilical vein ECs (HUVEC) for 8 hours did not alter the angiogenic process (i.e. EC branches) formed by HUVEC. In fact, even when 40 platelets were co-cultured with 1 HUVEC, no change in angiogenic performance was observed (Fig. 1A). This observation was somewhat surprising given the initial observation by Pipili-Synetos and colleagues in 1998, that platelets promoted the formation of capillary-like structures by HUVEC on Matrigel *in vitro*^38^. To address this disparity, we performed additional experiments with lower seeding densities of HUVEC (i.e. 2.5 × 10^3^ or 5 × 10^3^ cells/well) but again observed no difference in angiogenesis when platelets were added (data not shown). Because the Pipili-Synetos study presented data of HUVEC angiogenesis with and without platelets as ‘relative tube area’, rather than the number of EC branches presented here, we reanalysed our data via ImageJ using an threshold mask algorithm to determine well area covered by vascular tubes (i.e. relative tube area). As shown in Supplementary Fig. 2, even at a ratio of 1 HUVEC to 40 platelets, the relative tube area did not differ from HUVEC alone.

With an increasing interest in vasculogenic mimicry (VM) by cancer cells, we next examined whether platelets influenced VM formation by cancer cells *in vitro*. To this end, we undertook similar experiments to those described above using two human melanoma and two human breast cancer VM-competent cell lines (Fig. 1B, left hand images). In contrast to what we observed with the HUVEC, when C32 melanoma cancer cells were co-cultured with platelets a robust and reproducible inhibition of VM formation was observed. Video footage in the Supplementary Data captures this disruption of VM formation with increasing numbers of platelets. When 40 platelets were added to 1 cancer cell a ~90% reduction in VM formation was observed (Fig. 1B). To address whether platelets can also disrupt established VM networks, platelets were added to the VM assay 4 hours post C32 cancer cell seeding. Supplementary Fig. 3 shows that the addition of platelets to largely established VM structures caused a dissociation of the network suggesting that platelets can, at any time, interfere with the VM process. Interestingly, CHL-1 melanoma cells exhibited a greater resistance to the platelets and for the most part maintained VM competence. For both breast cancer cell lines (HS-578T and MDA-MB-231) platelets significantly inhibited VM formation (Fig. 1B, inhibition of ~56% and ~64%, respectively). Taken together, these results suggest that when cancer cells are exposed to increasing numbers of platelets, their ability to form VM structures, at least *in vitro*, is perturbed. To determine whether these changes in VM may be attributable to platelets directly affecting cancer cell survival, an alamarBlue viability assay was performed. Repeated experiments confirmed that MDA-MB-231 cancer cells co-cultured with platelets for up to 24 hours (ratio of 1:40) retained a viability equivalent to that of cancer cells alone.

### Mechanism of platelet action

**Figure 2:**
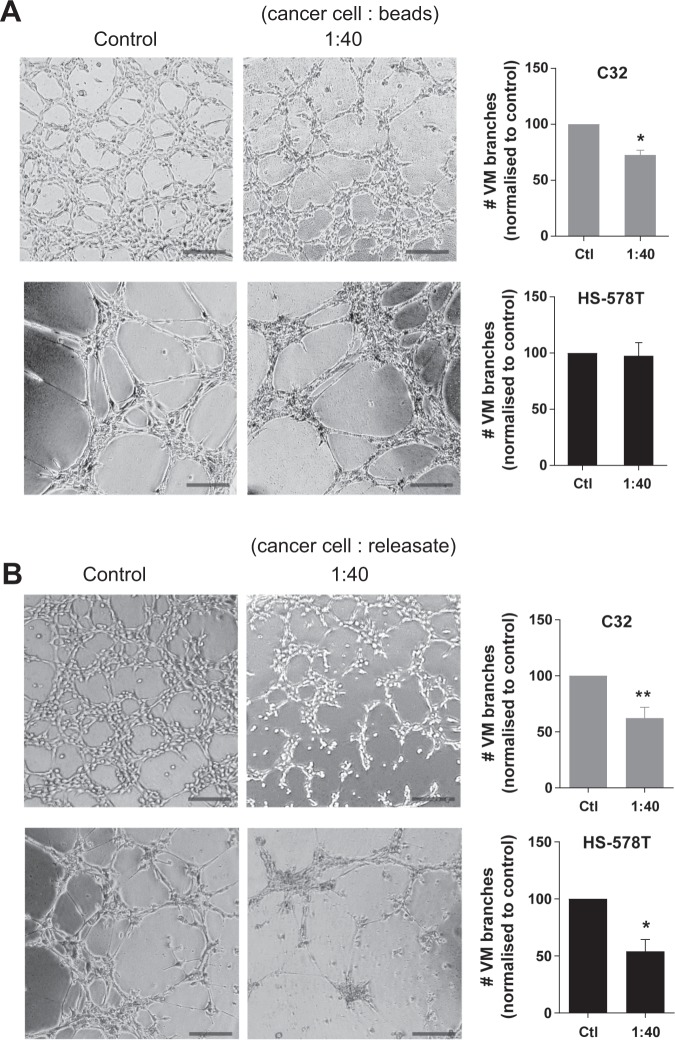
VM formation by cancer cells in the presence of platelet-sized beads and platelet releasates. In (**A**); representative images of C32 melanoma and HS-578T breast cancer cells undergoing VM in the presence of buffer control or platelet-sized beads (2 μm) at the indicated ratio (cells:beads). VM structures are expressed as mean ± SEM for n = 3 experiments. *p < 0.05 compared with buffer control, one-way ANOVA. Scale bar is 200 µm, original magnification 40x. In (**B**); C32 melanoma and breast cancer cells without and with co-culture of α-thrombin-activated platelet releasate at the indicated ratio (cells:supernatant) where the supernatant is the released contents from the respective number of platelets. Data are expressed as mean ± SEM from n = 3 experiments. *p < 0.05, **p < 0.01, one-way ANOVA. Scale bar is 200 µm, original magnification 40x.

To confirm that the effect of platelets on cancer cell VM was not mediated simply through spatial interference, we performed similar experiments in which inert platelet-sized (i.e. 2 µm) polystyrene microspheres were added to the VM assay. Figure 2A shows that when platelet-sized beads were added to either the C32 melanoma cells or HS-578T breast cancer cells at a ratio of 1:40 (cancer cell:beads), the beads were less effective in their inhibition of VM with only a 28% reduction in VM by the C32 melanoma cells and 2% reduction in VM by the HS-578T breast cancer cells.

We next investigated whether platelets inhibit VM formation via their release of a soluble factor from α-granules. To do this, purified platelets were activated with human α-thrombin, a known cancer cell produced platelet agonist which increases P-selectin expression (confirmation of activity shown in Supplementary Fig. 4) and causes degranulation^39,40^. The collected ‘releasates’ were then co-cultured with the either the C32 melanoma cells or HS-578T breast cancer cells at an approximate ratio of 1:40 (cancer cell:releasate volume) and the VM formation monitored as above. As shown in Fig. 2B, the releasate co-cultured with C32 melanoma cells and HS-578T breast cancers cells caused a 38% and 46% reduction in VM formation respectively. These results suggest that for platelet inhibition of VM to be most effective the platelets are ideally in close proximity, or in direct contact, with the cancer cells and that they release soluble factors.

### Platelets and cancer cell invasion

**Figure 3:**
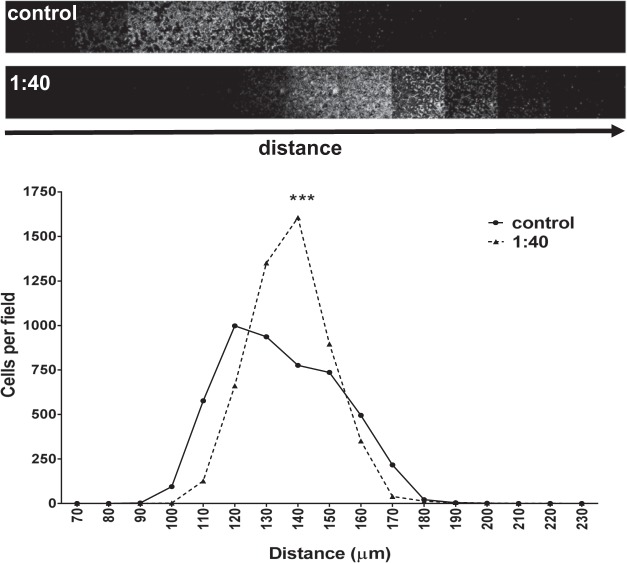
Platelets promote cancer cell invasion *in vitro*. MDA-MB-231 cells co-cultured without or with platelets (cells:platelets) were allowed to invade Matrigel covered Transwells in an inverse invasion assay. Following 3 days of invasion, cells were stained with propidium iodide and serial optical sections (10 μm intervals) were acquired via confocal microscopy. Magnified images from z = 12 sections are shown (top). Cell invasion was quantified as the number of cells over distance travelled and then normalized to control treated cells for each experiment. Data show mean ± SEM, n = 3, ***p < 0.001, two-way ANOVA.

Precisely what factors are released by platelets to perturb VM formation is yet to be determined, but we hypothesized that this disruption of VM network might serve as a means to permit the migration of cancer cells, i.e. metastasis. To address this, we performed an inverse invasion assay wherein C32 melanoma cells without or with platelets co-cultured at a ratio of 1:40 as above, penetrated through an extracellular matrix (Matrigel) towards 10% FCS as a chemoattractant. Figure 3 shows that the cancer cells seeded together with the platelets exhibited a greater invasive potential as demonstrated by them being able to migrate further through the matrix and towards the FCS gradient. Quantitation of cell number relative to distance travelled confirmed that the platelets significantly enhanced the number of cancer cells invading into the matrix as well as their distance travelled (Fig. 3, histogram).

### VM formation in melanoma of thrombocytopenic mice

**Figure 4:**
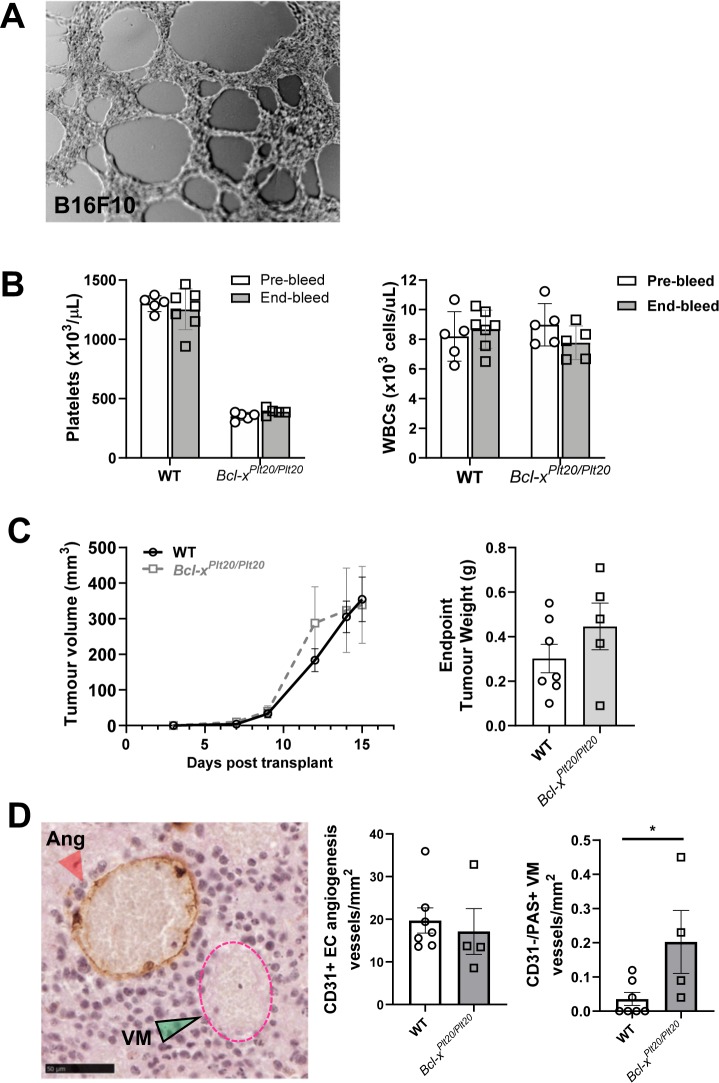
VM formation by B16F10 melanoma cells and influence of platelets *in vivo*. In (**A**); representative image of B16F10 melanoma cancer cells undergoing VM *in vitro* in Matrigel. In (**B**), circulating platelet and WBC counts in wildtype (WT) and *Bcl-x*^*Plt20/Plt20*^ mice prior to, and experimental end (open bars, pre-bleed at day -14, grey bars, end-bleed at day 15). In (**C**), caliper measurements of B16F10 tumour growth over time and final B16F10 tumour weights at experimental end (open symbols, WT mice; grey symbols, *Bcl-x*^*Plt20/Plt20*^ mice). In (**D**), representative image of CD31 and PAS stained B16F10 harvested tumour. CD31^+^/PAS^+^ EC-lined angiogenic structure (Ang, red arrow head) and CD31^−^/PAS^+^ VM structure (VM, green arrow head and pink dotted line). Scale bar is 50 µm. Corresponding quantification of the average angiogenic and VM structures per mm^2^ (open bars, WT mice; grey bars, *Bcl-x*^*Plt20/Plt20*^ mice). Data show mean ± SEM for n = 5–7 mice. *p < 0.05, unpaired *t*-test.

To investigate the contribution of platelets to VM formation *in vivo*, we used the syngeneic mouse model of B16F10 melanoma in the thrombocytopenic *Bcl-x*^*Plt20/Plt20*^ mice wherein platelet counts are reduced to ~25%^41^. First, we confirmed the ability of B16F10 melanoma cells to form VM using the *in vitro* angiogenesis assay (Fig. 4A). Next, we injected B16F10 cells into the flank of wildtype and *Bcl-x*^*Plt20/Plt20*^ mice. Figure 4B shows that the *Bcl-x*^*Plt20/Plt20*^ mice had reduced circulating platelet and white blood cell (WBC) counts both prior to, and at the conclusion of, the experiment. Figure 4C shows that neither tumour size (volume and weight) differed between the two groups.

To assess tumour vasculature, immunohistochemistry was performed on the harvested tumours. Histologically, EC-lined blood vessels (angiogenesis) are identified by their expression of CD31 while VM structures are CD31-negative but can be visualised using periodic acid-Schiff (PAS) reagent, which stains basement membranes rich in collagen and laminin^30^. Figure 4D shows that the tumours in the *Bcl-x*^*Plt20/Plt20*^ mice contained significantly more VM structures than their wildtype counterparts. No difference in CD31+ EC-lined tumour angiogenesis was observed between the two groups (Fig. 4D). No metastasis was detected in the lungs or livers of the mice (data not shown) and is consistent with this relatively short and subcutaneous B16F10 model^42,43^.

### Low-dose aspirin and breast cancer progression *in vivo*

**Figure 5:**
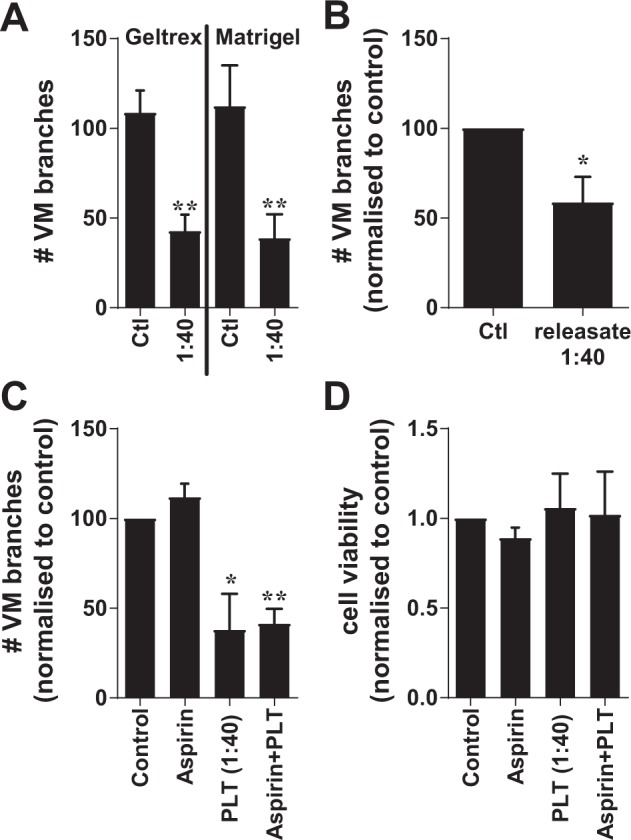
VM formation and survival assays with MDA-MB-231 cancer cells in the presence of platelets, platelet releasates or Aspirin. In (**A**); MDA-MB-231 breast cancer cells undergoing VM in Geltrex or Matrigel in the presence of buffer control (Ctl) or platelets at the indicated ratio (cells:platelets). VM structures are expressed as mean ± SEM for n = 4–5 experiments. **p < 0.01 compared with buffer control, paired t-test. In (B); MDA-MB-231 breast cancer cells undergoing VM without and with co-culture of α-thrombin-activated platelet releasate at the indicated ratio (1:40 releasate equivalent). Data are expressed as mean ± SEM from n = 3 experiments. *p < 0.05, paired t-test. In (C), MDA-MB-231 breast cancer cells co-cultured with Aspirin (100 μM), platelets at the indicated ratio (cells:platelets) or platelets pre-treated with Aspirin (100 μM) for 10 min prior to inclusion in the VM assay. VM structures are expressed as mean ± SEM for n = 6 experiments. *p < 0.05, **p < 0.01, one-way ANOVA. In (D), MDA-MB-231 cells cultured without or with Aspirin (100 μM), platelets (1:40 ratio) or Aspirin (100 μM) pre-treated platelets prior to cell viability being examined at 24 hours via alamarBlue. Results are expressed as mean ± SEM for n = 7 experiments.

To further investigate the contribution of platelets to VM formation *in vivo*, we undertook an orthotopic xenograft model of triple-negative breast cancer (TNBC) using the MDA-MB-231-LM2 cells (a variant of the MDA-MB-231 cell line modified with the a luciferase tag^44^). Our experimental plan was to inject the cancer cells as a bolus in growth factor-reduced Matrigel into mice with a subgroup of those mice given a daily gavage of low-dose aspirin (25 mg/kg) to inactivate the platelets. Prior to the *in vivo* experiments, *in vitro* experiments confirmed that platelets inhibit VM formation as equally in Matrigel as we had observed in Geltrex (Fig. 5A). We also confirmed that VM by MDA-MB-231 cells was inhibitable by the releasate of α-thrombin activated platelets (Fig. 5B) and  investigated whether exposure of MDA-MB-231 cells to aspirin alone would influence VM formation, it did not (Fig. 5C). Similarly, exposure of platelets to aspirin did not alter their inhibition of VM (Fig. 5C). The viability of these breast cancer cells was also not affected but exposure to aspirin or releasate over 24 hours (Fig. 5D).

**Figure 6:**
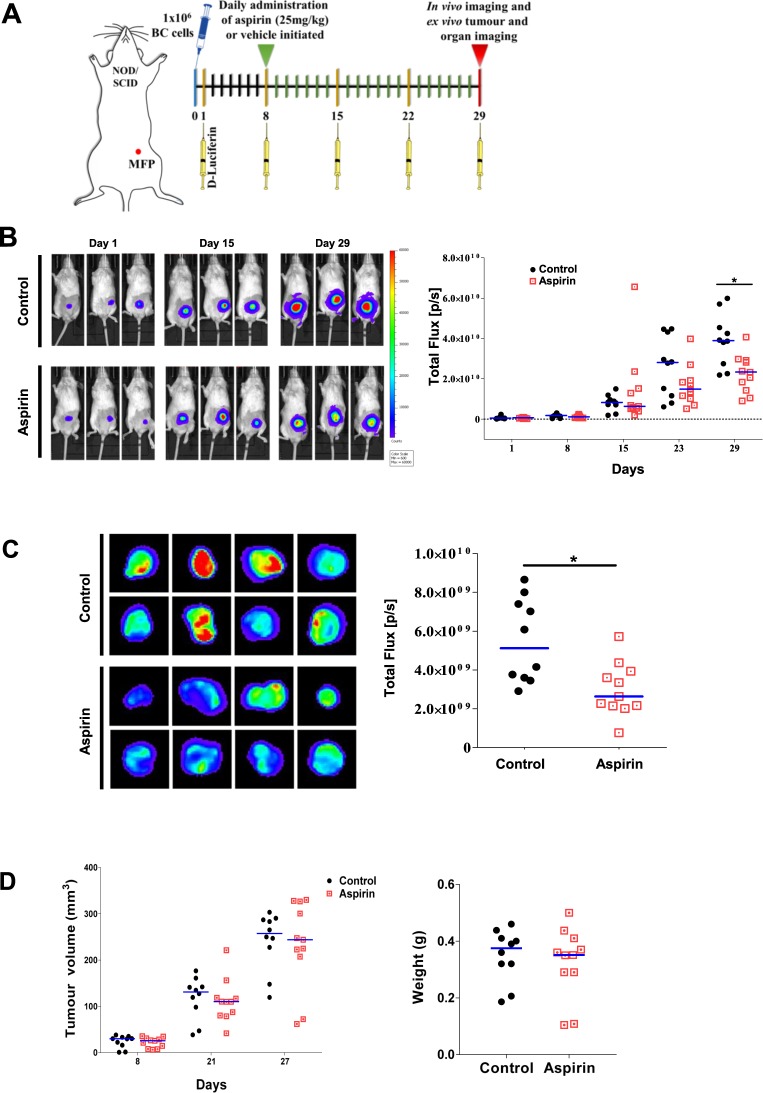
Aspirin reduces MDA-MB-231-LM2 breast cancer burden *in vivo*. (**A**) Schematic of experimental protocol. (**B**) Representative *in vivo* bioluminescence images of three control (vehicle) and three aspirin-treated mice (25 mg/kg) taken on day 1, 15, and 29. Bioluminescence images are shown as a heat map with blue representing low intensity; red representing high intensity. Corresponding quantification of bioluminescence in control (n = 10) and aspirin-treated mice (n = 11) is expressed as the total flux (photons/second) at each week post MDA-MB-231-LM2 cancer cell injection. Blue lines in graph represent the median value, *p < 0.05, Mann-Whitney U test. (**C**), Representative bioluminescence images of excised tumours shown as a heat map with corresponding quantification expressed as the total flux (photons/second). (**D**) Caliper measurements of tumour volume (mm^3^) in control and aspirin-treated mice measured on days 8, 21, and 27 post MDA-MB-231-LM2 cancer cell injection. Blue line represents the median. Tumour weights from control and aspirin-treated mice at experimental end (day 29 where blue lines in graph represent the median values.

With these results in hand, MDA-MB-231-LM2 cancer cells were injected into the mammary fat-pad (MFP) and tumour growth was monitored on day one after tumour cell injection, and weekly thereafter (days 8, 15, 22, and 29 post implantation) through bioluminescence imaging (Fig. 6A). As illustrated in Fig. 6B, quantification of the bioluminescence signal revealed a statistical difference in tumour burden between the control and aspirin-treated mice at day 29 post implantation. Heat maps of the excised tumours revealed lower bioluminescence readings from the aspirin-treated mice at experimental end point, day 29 (Fig. 6C). Interestingly, caliper measurements taken throughout the experiment, as well as final tumour weights, showed no significant differences in tumour volume (Fig. 6D,E).

**Figure 7:**
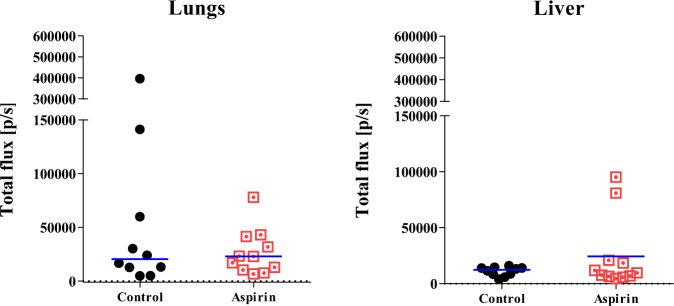
Aspirin treatment and breast cancer metastasis in mice On day 29 post MDA-MB-231-LM2 cancer cell injection into the mammary fat pads of mice treated without and with aspirin, the lungs and liver were harvested and imaged for bioluminescent cancer cell content. Metastatic events were quantified by measuring total flux (photons/second). Lines in graph represent the median values.

With Coupland and colleagues having shown that platelets assist breast cancer metastasis to the lung but not the liver^45^, these two organs were harvested from the aforementioned mice to examine their cancer cell content via luciferin-induced bioluminescence. Figure 7 shows that at experimental end, no significant difference could be detected in metastatic load in either the lungs or liver across the two groups of mice.

### Apoptotic potential of aspirin in breast cancer *in vivo*

**Figure 8:**
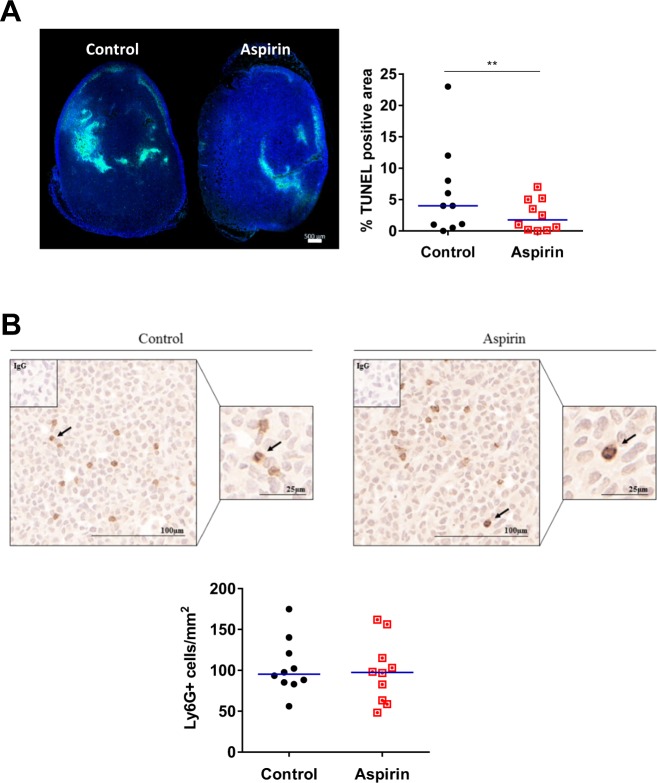
Analysis of tumour necrosis and neutrophil content in control versus aspirin-treated mice. (**A**) Representative images of TUNEL staining from tumours harvested from control and aspirin-treated mice. DAPI (blue) fluorescence detects nuclei and FITC (green) fluorescence indicates DNA fragmentation in cells. Scale bar is 500 μm. TUNEL positivity quantified as a percentage of total tumour area. Blue lines in graph represent the medians of n = 10 tumours per group. **p < 0.01, Mann-Whitney U test. In **(B**), representative images of Ly6G stained MDA-MB-231-LM2 xenograft tumours harvested from mice treated without and with aspirin. Black arrows indicate Ly6G+ neutrophils. Scale bars are 100 µm and 25 µm. Neutrophils quantified per whole tumour. Blue lines in graph represent the medians. Mann-Whitney U test.

To determine why the aspirin-treated mice harboured tumours of the same overall size (i.e. tumour volume, Fig. 6D) but reduced bioluminescence (i.e. cancer cell content, Fig. 6C) a histological assessment of the tumours was performed via TUNEL assays to detect apoptotic cells. As shown in Fig. 8A, all tumours harvested from the mice contained necrotic regions with a significant reduction in apoptosis detected in the aspirin-treated mice, an observation that correlates with reduced cancer cell burden by these mice.

### Ly6G+ cells in TNBC *in vivo*

In an attempt to further explain why the overall tumour size and weight did not differ between the two groups of mice, we examined whether other cells, such as scavenging neutrophils, may be more or less abundant. As shown in Fig. 8B, Ly6G+ neutrophils could be identified in tumours from both control and aspirin-treated mice with no discernible differences.

### Tumour vascularity; angiogenesis and VM

**Figure 9:**
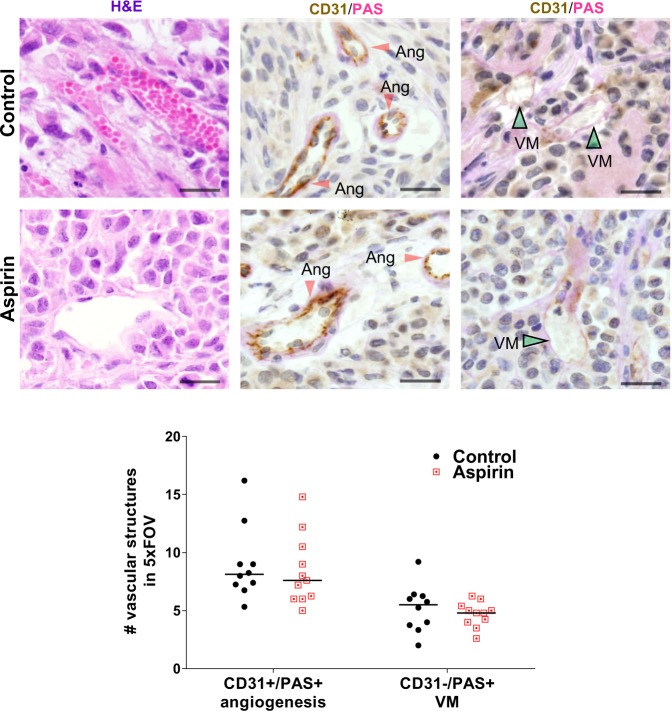
Angiogenesis and VM structures in MDA-MB-231-LM2 breast cancer tumours. (**A**) Representative images of CD31 and PAS stained MDA-MB-231-LM2 xenograft tumours harvested from mice treated without and with aspirin. Red arrow heads identify CD31^+^/PAS^+^ staining for EC-lined angiogenic structures (Ang) and green arrow heads identify CD31^−^/PAS^+^ staining for VM structures (VM). Corresponding quantification of the average angiogenic and VM structures in 5 fields of view (FOV), in tumour sections from control and aspirin-treated mice. Lines represent the medians and scale bar is 20 µm. Mann-Whitney U test.

To assess the impact of aspirin treatment on angiogenesis and VM in breast cancer, immunohistochemistry was performed on the harvested tumours. Quantitation of angiogenic and VM structures revealed no significant difference between the tumours from control and aspirin-treated mice (Fig. 9). Notably, in the tumours of control and aspirin-treated mice, ~64% of the vascular structures were EC-lined (CD31+/PAS+) while the remaining ~36% were VM structures (CD31−/PAS+). These results suggest that at this single time point (day 29 of MDA-MB-231-LM2 breast cancer growth in NOD/SCID mice) there is no discernible difference in tumour vascularity as a result of low-dose aspirin treatment.

### Gene expression profile of VM by TNBC cells *in vivo* following aspirin-treatment

**Figure 10:**
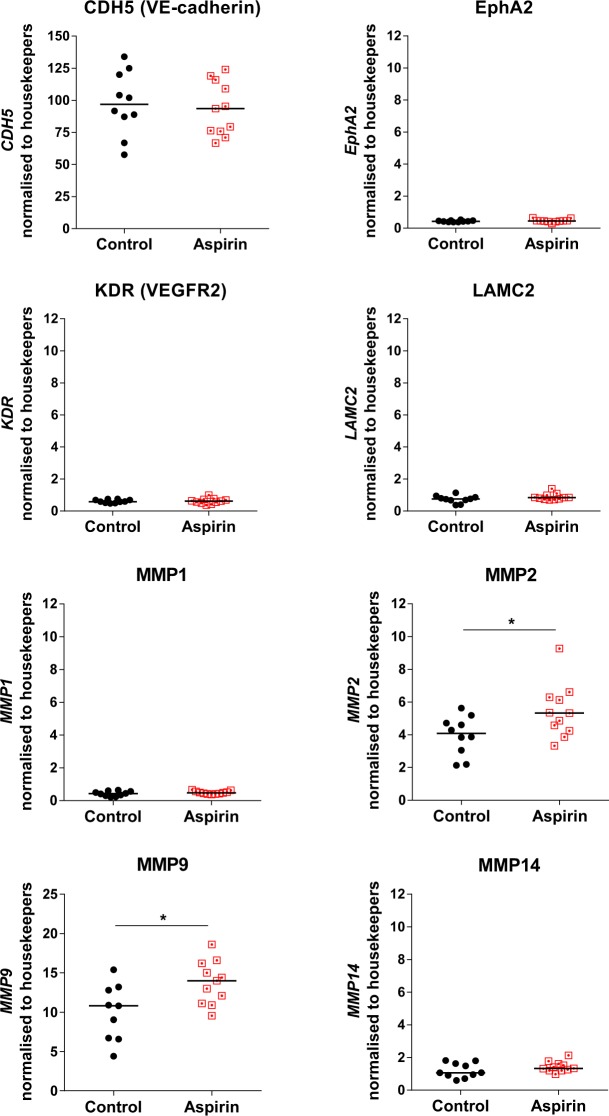
Aspirin treated mice with breast cancer have altered gene expression *in vivo*. Gene expression was determined via RT-qPCR of MDA-MB-231-LM2 breast cancer tumours harvested from mice treated without and with aspirin at experimental end. Scatter dot plot showing the mRNA gene expression of eight VM-associated genes; *CDH5*, *EPHA2*, *KDR*, and *LAMC2*, *MMP1*, *MMP2*, *MMP9*, and *MMP14* normalised to housekeeping genes (*GAPDH*, *ACTB*, and *CYCA*). Blue lines in the graph represent the median values, *p < 0.05, Mann-Whitney U test.

Finally, we assessed whether aspirin treatment of the mice affected the gene expression by the human breast cancer cells. Of particular interest was a select group of genes documented to be upregulated with VM occurrence^29,30^; namely genes associated with vascular structures (*CDH5* (vascular endothelial (VE)-cadherin), *EPHA2*, *KDR* (vascular endothelial growth factor receptor 2,VEGFR2), genes associated with the extracellular matrix (laminin, *LAMC2*) and key matrix-metalloproteases (MMPs) *MMP1*, *MMP2*, *MMP9*, and *MMP14*. To identify only the changes caused by aspirin on the human cancer cells, we constructed primer sequences to detect only human genes (Supplementary Table 1). As shown in Fig. 10, *CDH5* (VE-cadherin) was the most abundantly expressed gene of those tested and did not differ between the tumours harvested from control or aspirin-treated mice. Similarly, gene expression of *EPHA2, KDR, LAMC2, MMP1*, and *MMP14* did not vary between tumours of mice treated without and with aspirin. In contrast, expression levels of both *MMP2* and *MMP9* were significantly higher in tumours excised from the aspirin-treated mice (Fig. 10).

## Discussion

This study provides several novel insights into the role of platelets in cancer. First, we reveal with *in vitro* angiogenesis assays that platelets do not perturb capillary-like tube formation by HUVEC, but they do inhibit VM by human cancer cells. This inhibition of VM was observed across three out of four cancer cell lines (C32 melanoma as well as HS-578T and MDA-MB-231 breast cancer cells) with one melanoma cell line (CHL-1) exhibiting only a trend for VM inhibition by the platelets. We observed a consensus for 20–40 resting platelets per cancer cell to effectively inhibit VM formation by 56–88% and that platelet sized beads and the releasates from activated platelets could also inhibit VM formation. Our observation that whole platelets were more effective at blocking VM formation than either beads or releasate alone, suggests that direct contact between the platelet and the cancer cell maximises the opportunity for one or more anti-VM factors to be released from the platelets. Our *in vitro* inverse invasion assay suggests that platelets also promote cancer cell migration which supports the recent publication by Lucotti and colleagues of platelets orchestrating an intravascular metastatic niche to promote tumour cell seeding^43^. Interestingly, the pre-treatment of platelets with aspirin did not prevent them from inhibiting VM *in vitro*. One explanation for this may be that platelets inherit their functions from their megakaryocyte precursors^41,43^ and that exposure of pre-formed platelets to aspirin cannot influence their VM-inhibiting functions. This concept is supported by documentation that aspirin can modulate many megakaryocyte genes (e.g. integrins and transcription factors) resulting in platelets containing an ‘aspirin response gene signature’ which influences their function *in vitro* and *in vivo*^46–48^.

Our *in vitro* angiogenesis results concur with those of Kuznetsov and colleagues who elegantly showed that resting/unactivated platelets are not pro-angiogenic for HUVEC *in vitro*^49^. In contrast, Pipili-Synetos and coworkers claimed that resting platelets promoted angiogenesis by HUVEC *in vitro*^38^. Notably, their study did not provide evidence that their platelets were not activated (e.g. low in P-selectin expression). The importance of platelet activation for angiogenesis is supported by Etulain and colleagues who published that releasates from thrombin-activated platelets are proangiogenic for human microvascular ECs *in vitro*^27^; notably, unactivated platelets were not examined. Here we reveal an effect of platelets on a second form of tumour vasculature, vasculogenic mimicry, with platelets actively disassembling VM structures formed by cancer cells. This interference was observed (to varying degrees) across two human breast cancer lines and two human melanoma cell lines. Our observation that VM disassembly by platelets is most effective when platelets come into direct contact with the cancer cells supports the discovery by Pang and colleagues of thrombin-activated platelet membranes needing to bind to the MDA-MB-231 cancer cells via P-selectin and GPIIb/IIIa to promote cancer cell migration and metastasis *in vivo*^50^. Whether platelets become activated as they inhibit VM formation is still to be determined. The divergent nature of platelet function *in vitro* has been published by Battinelli and colleagues who showed that human platelets activated with adenosine diphosphate stimulated the release of pro-angiogenic VEGF which promoted HUVEC angiogenesis *in vitro*, whereas platelets activated with thromboxane A2 released the anti-angiogenic endostatin resulting in inhibition of angiogenesis by HUVEC^51^. Whether these pathways, or others, are involved in the regulation of VM by cancer cells is yet to be determined. With increasing interest in targeting VM to combat cancer progression^31,52^, the current study also reveals that co-culture of platelets with cancer cells did not influence cell viability but did promote cancer cell migration. This observation is also supported by Pang and coworkers who published that when MDA-MB-231 cells come into direct contact with the membranes of thrombin-activated platelets, the cells adopt a Matrigel-degrading phenotype via P-selectin and GPIIb/IIIa^50^. In addition, Labelle and colleagues published that platelet-derived TGFβ and direct platelet-tumour cell contacts synergistically activate the TGFβ/Smad and NFκB pathways in cancer cells, resulting in their transition to an invasive mesenchymal-like phenotype and enhanced metastasis *in vivo*^53^. Based on these observations, we speculate that platelets may also actively dissolve VM structures to promote metastasis. Precisely what soluble factor/s are being released by the platelets, or whether platelet-derived extracellular vesicles perturb VM formation and promote cancer cell migration is yet to be determined. Our discovery that platelets have no effect on angiogenesis but actively disassemble VM structures *in vitro* and *in vivo* may provide important information on the differences between these two processes of tumour vascularisation for therapeutic purposes^52^.

While mouse models have clearly demonstrated that platelet depletion attenuates cancer metastasis^45,54^ the use of aspirin to inactivate platelets has also yielded promising results^49,55^. For example, administration of medium-dose aspirin (75 mg/kg, equivalent to 360 mg human analgesic) in a MDA-MB-231 xenograft model showed that aspirin prevented tumour growth and induced caspase-3-mediated apoptosis of the cancer cells^55^. Similarly, low-dose aspirin (10–25 mg/kg, equivalent to ≤150 mg human anti-platelet/analgesic) in animal models of neuroblastoma and colorectal cancer reduced tumour growth, metastasis and chemoprotection^56–59^. The current study has investigated the effects of oral low-dose aspirin (25 mg/kg) on the MDA-MB-231-LM2 xenografted mice and revealed reduced cancer cell burden with decreased necrosis, but no effect on overall tumour size, neutrophil content or tumour vascularisation. Coupland and colleagues reported that in the lungs, platelets play a significant role in anchoring the tumour to the endothelium against the relatively high inherent blood flow velocity. In the liver however, where the blood flow is comparatively slow due to hepatic vessel anatomy, interactions between endothelial cell and tumour cell adhesion proteins are sufficient to arrest tumour cells enabling their subsequent extravasation, with platelet assistance not being required^45^. While we observed no significant difference in overall metastases between our groups of mice, a slight reduction in lung metastasis was detected and thus supports the aforementioned Coupland *et al.* study. A more recent study by this same group revealed the importance of cell-cell contact between thrombin-activated platelets and MDA-MB-231 cells to promote lung metastasis *in vivo*^50^. The effect of aspirin on cancer progression in humans is also unclear with Marshall and co-workers documenting that long-term daily aspirin use was associated with an increased risk of estrogen receptor/progesterone receptor (ER/PR)-negative breast cancer but decreased risk of ER/PR-positive breast cancer^60^. More recently, Shaio and colleagues showed that aspirin improved the 5-year disease-free survival rate for patients with Stage II-III TNBC^61^. In conflicting observations by others, low-dose aspirin (100 mg every other day) showed no preventative effect on breast cancer^61,62^. Clearly, a better understanding of the effect of aspirin across the breast cancer subgroups is warranted.

Immunohistochemical staining to identify EC-lined tumour vasculature (CD31+/PAS+) and VM (CD31−/PAS+) vascular structures showed no differences in tumour vasculature in the breast cancers harvested at experiment completion from mice without or with aspirin. However, the administration of aspirin to tumour bearing mice did influence the known VM gene signature^52^ by the cancer cells. Upon examination of VE-cadherin, ephrin A2, VEGFR2, laminin 5γ2, MMP1, MMP2, MMP9 and MMP14 by the human cancer cells, we made the surprise finding that of the genes investigated, VE-cadherin (*CDH5*) was the most highly expressed. Normally restricted in expression by endothelial cells^63,64^, *CDH5* is gaining recognition as a prominent factor involved in VM formation by aggressive cancer cells^52^ and its downregulation in melanoma results in a loss of VM formation^65^. We observed no differences in expression of *CDH5* in the tumours harvested from mice treated without or with aspirin, however post-translation modifications of VE-cadherin also trigger changes in vascular integrity and leukocyte trafficking^66^. Congruent with this, Delgado-Bellido and colleagues published that phosphorylation of VE-cadherin residue Y658 by focal adhesion kinase (FAK) promoted VM formation by melanoma cells via kaiso-dependent gene expression^67^. Two additional genes known primarily for their contribution to angiogenesis, VEGFR2 (*KDR*) and Ephrin A2 (*EPHA2*), were lowly expressed by the human cancer cells and did not change in response to aspirin treatment. Of the four MMPs investigated, *MMP1* and *MMP14* were the lowest in expression and did not change in response to aspirin treatment. In contrast, *MMP2* and *MMP9* were highly expressed and these levels were further elevated in the mice treated with aspirin. The proteinases MMP2 and MMP9 are associated with cancer progression with known roles in degradation and remodelling of the surrounding extracellular matrix (ECM) to facilitate tumour angiogenesis, invasion and metastasis^68,69^. These MMPs exhibit elevated expression at the leading edge of invasive tumours^70^. Our observation of aspirin-treated mice harbouring cancer cells with increased MMP2 and MMP9 gene expression is intriguing and whether this has resulted from aspirin acting on the platelets or the cancer cells themselves is still to be determined. The latter is possible with aspirin known to bind to IκB kinase (IKK) β and prevent NFκB activation both *in vitro* and *in vivo*^71^ to alter the transcriptome of a diverse array of proteins that stimulate proliferation, migration and survival in cancer^24^. However, with Shi and colleagues showing that prostate cancer cells treated with aspirin significantly reduce MMP9 activity without any effect on MMP2 activity^72^, and an aspirin analogue ATL-1 capable of inactivating FAK in ECs^73^, the effect of aspirin on cancer cells is complex and yet to be fully elucidated.

In summary, this study suggests that co-culture of HUVEC with unstimulated platelets does not influence angiogenesis *in vitro*. In contrast, unstimulated platelets robustly and reproducibly inhibit VM formation by multiple cancer cell lines *in vitro*. This anti-VM effect by platelets was observed not only during the initial formation of VM structures but also as an active disassembly of existing VM structures. We observed that this was achieved by whole platelets and to a lesser extent by thrombin-induced platelet releasates. The inability of aspirin to block platelet inhibition of VM suggests a pathway/process independent of COX and likely via gene regulation of the megakaryocytes during platelet biogenesis. A role for platelets in VM formation was supported *in vivo* with the B16F10 tumours in the thrombocytopenic *Bcl-x*^*Plt20/Plt20*^ mice exhibiting increased VM content when compared to wildtype controls. Interestingly, our attempt to inactivate platelets and perturb breast cancer growth *in vivo* via administration of low-dose aspirin resulted in a significant reduction in tumour burden and corresponded with a decrease in necrotic regions within the tumours without discernible difference in tumour vascularization (angiogenesis or VM) or metastasis at experimental end. Taken together, the *in vitro* results suggest that targeting platelets may prevent the disassembly of VM structures, prevent cancer cell migration and perturb breast cancer burden *in vivo*. Precisely how this inhibition of platelet function in cancer is brought to fruition is yet to be determined, but unlikely to be made possible from the administration of aspirin alone.

## Methods

### Ethics statement

The collection of primary HUVEC and platelets was approved by the Human Research Ethics Committees of the Royal Adelaide Hospital (RAH) and the University of South Australia, Adelaide, South Australia. Informed written consent was obtained from subjects in accordance with the ‘Declaration of Helsinki’. Animal experiments were approved by the Animal Ethics Committee of SA Pathology or the Walter & Eliza Hall Institute and conform to the guidelines established by the ‘Australian Code of Practice for the Care and Use of Animals for Scientific Purposes’.

### Statistical analysis

Data were expressed as mean ± standard error of the mean (SEM). Statistical analyses and significance were calculated by Student’s *t* test, Mann Whitney U test or ANOVA to determine statistical significance using GraphPad PRISM software (San Diego, CA, USA). In all comparisons, *p* < 0.05 was considered statistically significant.

### Platelet purification and validation

Blood from healthy donors (aged 18–65 years, non-smokers and aspirin/NSAIDs free for at least 1 month) was collected into acid-citrate dextrose (ACD, pH 4.5) anticoagulant and platelets purified as described elsewhere^74^. Briefly, blood was collected from single donors, rested at RT for 15 min prior to 180 g centrifugation, the platelet-rich-plasma was then centrifuged at 1,100 g for 15 min and the platelet pellet was resuspended gently with Tyrode’s platelet wash buffer (137 mM NaCl, 2.7 mM KCl, 1mM MgCl_2_.6H_2_O, 12 mM NaHCO_3_, 0.4 mM NaH_2_PO_4_.H_2_O, 3.7 mM HEPES, pH 7.4) containing 1.78U/mL Apyrase (BD Biosciences, San Jose, CA, USA). The platelet suspension was incubated at 37 °C for 5 min, centrifuged at 1,100 × g for 15 min then resuspended in Tyrode’s platelet resuspension buffer (Tyrode’s wash buffer, 3 mM CaCl_2_) containing 0.178U/mL Apyrase. Platelets were counted using a Sysmex XE-5000 differential analyzer (Sysmex Corporation, Kobe, Japan) and validated for purity and low-level activation via flow cytometric analysis. Platelets were stained with anti-CD42b and anti-CD62P or isotype-matched control antibody (all BD Biosciences, Ann Arbor, MI, USA) for 15 min with samples processed by a BD Accuri C6 flow cytometer with subsequent analyses performed on FCS Express 6 cytometry software (De Novo Software, Glendale, CA, USA).

### Collection of activated platelet releasate

Platelet releasates were prepared as previously described^53^ with washed platelets at ~1 × 10^8^/mL activated by 0.5IU human α-thrombin (Hyphen Biomed, Neuville-sur-Oise, France) for 10 min. Platelet activation was assessed by expression of CD62P and releasates collected following centrifugation (2,800g for 10min) and stored at −20 °C until used.

### Cell viability assay

MDA-MB-231 cells were seeded at 1.5 × 10^4^ cells/well in 96-well plate 24 hours prior to treatment without or with 100 μM Aspirin (Sigma, diluted in 100% ethanol (Sigma)) for up to 24 hours. Similarly, MDA-MB-231 cells were treated with platelets ± Aspirin (100 μM, 10 min prior to adding platelets to wells) at a 1:40 ratio of cancer cells to platelets. Cells were incubated at 37 °C, 5% CO_2_ for 24 hours. All treatments were performed in triplicate. AlamarBlue Cell Viability Reagent (Thermo Fisher) was added at 100 μl/well and incubated at 37 °C for 60 min prior to being read at 590 nm (FLUOstar Omega microplate reader (BMG Labtech, Mornington, Vic., Australia)).

### Angiogenesis and VM assays

HUVECs were isolated from human umbilical cords by collagenase digestion as previously described elsewhere^75^, were cultured in HUVEC media (M199 media (Sigma Aldrich, St. Louis, MO, USA) supplemented with 20% fetal bovine serum (FBS, Bovogen, Keilor East, Vic, Australia), Penicillin/Streptomycin (Life Technologies, Carlsbad, CA, USA) and non-essential amino acids (Sigma Aldrich)) and were used for no more than two passages. Human melanoma cell lines (CHL-1 and C32), human breast cancer cell lines (MDA-MB-231 and HS-578T) and mouse melanoma cell line (B16F10) were from American Type Culture Collection (ATCC) or gifted from Associate Professor Jeff Holst (University of New South Wales, Sydney, Australia) and MDA-MB-231-LM2 cells were kindly provided by Prof. Joan Massagué (Sloan-Kettering Institute for Cancer Research, New York, NY, USA). The melanoma cell lines (CHL-1 and C32) were cultured in RPMI media (Life Technologies) with 10% FBS and breast cancer cell lines (MDA-MB-231/LM2 and HS-578T) cell lines and mouse B16F10 cell line were cultured in DMEM media (Life Technologies) with 10% FBS.

*In vitro* angiogenesis assays of HUVEC or cancer cells was performed using Geltrex (Thermo Fisher Scientific, Waltham, MA, USA) or Matrigel (BD Biosciences) in the Angiogenesis µ-slides (Ibidi, Munich, Germany). Briefly, cells were seeded on a layer of Geltrex at a density of 1 × 10^4^ HUVEC; 1.3-2 × 10^4^ MDA-MB-231, HS-578T, C32, CHL-1 or B16F10 cells without or with platelet concentrations adjusted with Tyrode’s wash buffer to result in a platelet:cell ratio of 1:2, 1:5, 1:10, 1:20, and 1:40 (cells: platelets). Images covering the entire well were captured after 4–6 hours using an inverted imaging microscope (EVOS XL, Life Technologies), merged using Adobe Photoshop and tube-like structures manually counted using Image J software Cell Counter plugin (1.48pv, NIH, Bethesda, MD, USA). Percent relative tube area was also calculated for the HUVEC angiogenesis assays using a threshold mask on the capillary-like tubes across an entire well and measuring the percent area covered. In similar experiments, platelet-sized beads (Polysciences Inc., Warrington, PA, USA) or releasates collected from α-thrombin activated platelets were added to equal either platelet number or representative volume. In other experiments, platelets were pretreated ± 100 μM of Aspirin (or equivalent ethanol as vehicle control) 10 min prior to being combined with MDA-MB-231 at 1:40 ratio then seeded into the aforementioned VM assay. Representative videos of C32 melanoma cells treated without and with platelets at increasing ratios to cancer cells (i.e. cancer cell:platelet of 1:2, 1:5 and 1:20) over 8 hours via disk confocal live microscopy (CV100, Olympus, Tokyo, Japan). For established VM experiments, 2 × 10^4^ C32 cells were seeded, VM formation imaged at 4 hours prior to control buffer or platelets added at 1:40 ratio of cells:platelets and imaged again after 2 hours. Vascular-like structures were defined as multi-cellular elongated arrangements tightly aligned and extending between collections of cells as previously described^76^.

### Inverse invasion assay

Adapted from Hennigan *et al*.^77^, 100 µl of growth factor-reduced Matrigel diluted 1:1 in cold PBS was added into an 8.0 µm pore sized Transwell (Corning Inc., NY, USA) and allowed to set. Transwells were then inverted and 4 × 10^4^ MDA-MB-231 cells were seeded onto the underside of the membrane. Four hours later, the unbound cells were rinsed and Transwells immersed right-way up in serum-free HUVEC media without or with 16 × 10^6^/mL platelets and 10% fetal calf serum (FCS) was added to the upper chamber as chemoattractant, and cells allowed to migrate upward into the Matrigel for 48 hours. Transwells were paraformaldehyde fixed, RNAse-treated (100 µg/ml, Thermo Fisher) and stained with 0.05 mg/ml of propidium iodide (Thermo Fisher); all steps were carried out for 30 minutes with two PBS washes between each. Transwells were imaged at fixed intervals (10 μm) starting at the membrane and in a direction towards the chemoattractant using z-stack setting of Zeiss LSM 700 confocal microscope with a 20x objective (Carl Zeiss AG, Oberkochen, Germany). Cells from 3 fields of view per slice were quantified using ImageJ software, through threshold adjustment and counting particles (cells), this was then averaged.

### B16F10 mouse model of melanoma

For syngeneic studies, 7–8 week-old female or male, wildtype or *Bcl-x*^*Plt20/Plt20*^ mice^41^ on C57BL/6 background were used^78^. 1 × 10^6^ B16F10 cells in 50% growth factor-reduced Matrigel were injected subcutaneously into the flank. Caliper measurements of the tumours were taken every 2–3 days and after the animals were euthanized, their primary tumour, lungs and liver were harvested for histology. Blood was collected pre- and post- experiment from the retro-orbital sinus into Microtainer tubes containing EDTA, and circulating platelet and white blood cells (WBC) counts were performed using an ADVIA 2120 haematological analyser (Seimens, Munich Germany). Mice were rested for 2 weeks following bleeding prior to tumour cell injection.

### Preparation and administration of aspirin to mice

The mouse dose equivalent to 100–150 mg/60kg human low dose aspirin was calculated as *Human equivalency dose (HED) = animal dose (mg/kg)* × *(Animal Km)/(Human Km)*, where mouse Km factor is 3, and human Km factor is 37^79^. Aspirin (Sigma Aldrich) was prepared at 2.5 mg/mL to administer 10 µl/g body weight to deliver a dose of 25 mg/kg daily via oral gavage, with control mice receiving the same volume of corn oil (Sigma Aldrich).

### Orthotopic mouse model of breast cancer

For orthotopic studies, 5–6 week-old female mice were used and anesthetised before injections of 1 × 10^6^ MDA-MB-231-LM2 cells in 50 μl of 50% Matrigel (BD Biosciences) into the fourth mammary fat pad. Four weeks following inoculation, bioluminescence imaging was performed using the Xenogen IVIS-100 imaging system (Perkin Elmer, Waltham, MA, USA). Mice were injected intraperitoneally with 30 mg/ml of D-Luciferin (in PBS, Cayman Chemical, Ann Arbor, MI, USA) 10 min before imaging. Dorsal images of the primary tumour were collected before the animals were humanely killed and their primary tumour, lung and liver harvested for *ex vivo* imaging. Photon emission was quantified using the Living Image Software (Perkin Elmer).

### Histology and immunohistochemistry staining on tumours

Primary tumours were fixed in 10% buffered formalin for 24 hours before processing and embedding in paraffin. Sections (4 µm) were cut and subjected to heat-induce epitope retrieval (microwaved at 900W for 4 min, then 350W for 15 min) in 10mM citrate buffer pH 6.0 (for CD31 and Ly6G staining). Sections were allowed to cool to RT and quenched with 1–3% H_2_O_2_ prior to incubation with anti-CD31 antibody (1:250, Bethyl lab, Montgomery, TX, USA), an anti-Ly6G antibody (1:1000, BioLegend, San Diego, CA, USA) or an isotype IgG control overnight at 4 °C. Sections were then incubated for 30 min with avidin-biotinylated–horseradish peroxidase complex (Vectastain Elite ABC kit, Vector Laboratories, Burlingame, CA, USA) and visualized using peroxidase substrate solution (ImmPACT™ DAB, Vector Laboratories). Ly6G-stained sections were immediately counterstained with Ehlrich’s hematoxylin and mounted in DPX, while CD31-stained sections were further stained using a PAS staining kit from Merck Millipore (Burlington, MA, USA) according to manufacturer’s instructions before counterstaining and mounting. Stained sections were imaged using an Olympus BX40 microscope fitted with a DP70 digital camera and operated through the Olympus Image Analysis Cell B software program (Olympus). Ly6G-stained slides were scanned by the whole slide image (WSI) scanner (Hamamatsu NanoZoomer Slide scanner) and quantitated via ImageJ software. EC-lined blood vessels (CD31+/PAS+) and VM structures (CD31−/PAS+) were defined by the presence of RBCs or WBCs in the lumen. Structures were manually quantified in 4–6 fields of view (FOV) per tumour using ImageJ counter plugin software, and represented as average structures/FOV.

### TUNEL assay

Sections of formalin-fixed, paraffin-embedded tumours were stained with (fluorescein-labelled) TUNEL assay (Roche Diagnostics, Indianapolis, IN, USA) as per manufacturer’s instructions prior to mounting with ProLong™ Diamond Antifade Mountant with DAPI (Thermo Fisher Scientific, Waltham, MA, USA)), imaged via the Zeiss Axio Scan.Z1 and Zen Blue software (Carl Zeiss, Jena, Germany)) and analysed using ImageJ software. Positive TUNEL area was calculated as a percentage of total tumour area.

### RNA extraction, reverse transcription and quantitative PCR (qPCR)

RNA was extracted from cell pellets using the RNeasy Micro Plus kit (Qiagen, Hilden, Germany) according to the manufacturer’s protocol. Reverse transcription was performed on 1μg of purified RNA using a Superscript III enzyme (Life Technologies) following manufacturer’s instructions. Primers for PCR and qPCR (Supplementary Table 1) were either sourced from the literature or designed using Primer Blast (NCBI), synthesised (GeneWorks, Hindmarsh, SA, Australia) and validated for species specificity with Primer Blast (NCBI). qPCR was performed using QuantiTectTM SYBR Green master mix (Qiagen) on a Rotor-Gene thermocycler (Corbett Research, NSW, Australia) with reaction parameters: 15 minutes at 95 °C, then cycling of 10 seconds at 95 °C, 20 seconds at 57–60 °C, 30 seconds at 72 °C; for 45 cycles followed by a melt phase. Relative gene expression levels were calculated using the comparative quantitation method available in the Rotor-Gene Software (Corbett Research) and normalised against housekeeper genes *GAPDH*, *ACTB*, and *CYCA* validated using the geNorm algorithm (M value < 1.5).

## Supplementary information

Supplementary Information

.Supplementary Video 1

Supplementary Video 2

Supplementary Video 3
