# Supplementary material for: Platelets disrupt vasculogenic mimicry by cancer cells
Source: Sci Rep. 2020 Apr 3;10:5869. doi: 10.1038/s41598-020-62648-x (PMC7125143; doi:10.1038/s41598-020-62648-x)
Supplement: Supplementary file 1 — Supplementary Information [file 41598_2020_62648_MOESM1_ESM.pdf]

Platelets disrupt vasculogenic mimicry by cancer cells

Carmela Martini[1, 2], Emma J. Thompson[1], Stephanie R. Hyslop[3], Michaelia P. Cockshell[1], Brian J. Dale[2], Lisa M. Ebert[1], Anthony E. Woods[2], Emma C. Josefsson[3,4], Claudine S. Bonder[1,5]*.

[1] Centre for Cancer Biology, University of South Australia and SA Pathology, Adelaide, SA, Australia.
[2] School of Pharmacy and Medical Sciences, University of South Australia, Adelaide, SA, Australia.
[3] The Walter and Eliza Hall Institute of Medical Research, Parkville, VIC, Australia.
[4] Department of Medical Biology, The University of Melbourne, Melbourne, VIC, Australia.
[5] Adelaide Medical School, University of Adelaide, Adelaide, SA, Australia.

*Correspondence to claudine.bonder@unisa.edu.au

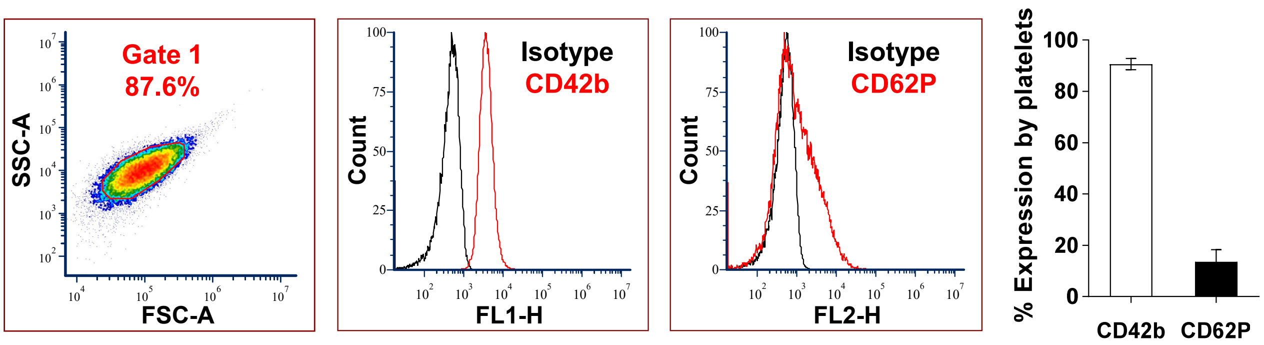

Supplementary Figure 1. Validation of platelet isolation and purification

Platelets purified from healthy donors analysed using flow cytometry with representative plots showing gated population which was stained for expression of CD42b and CD62P. Percentage of platelets stained positive and expressed as mean ± SEM. Results are pooled from 7 separate experiments from different platelet donors.

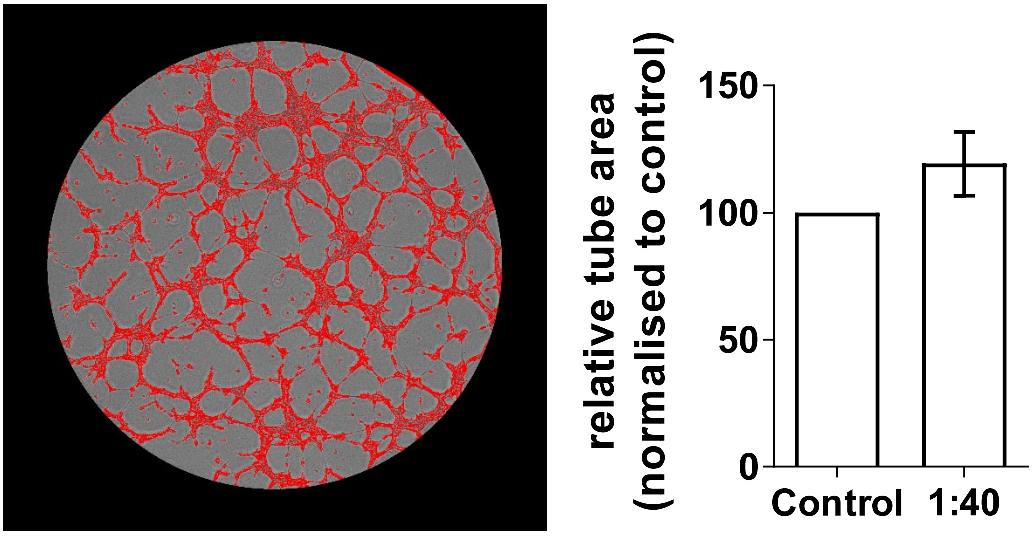

Supplementary Figure 2. Analysis of percent relative tube area for HUVEC angiogenesis exposed to platelets.

Representative images of HUVEC cells co-cultured with platelets (ratio of 1:40 ECs:platelets). Data are expressed as mean ± SEM with ImageJ threshold analysis results pooled from 5 separate experiments. Paired t-test.

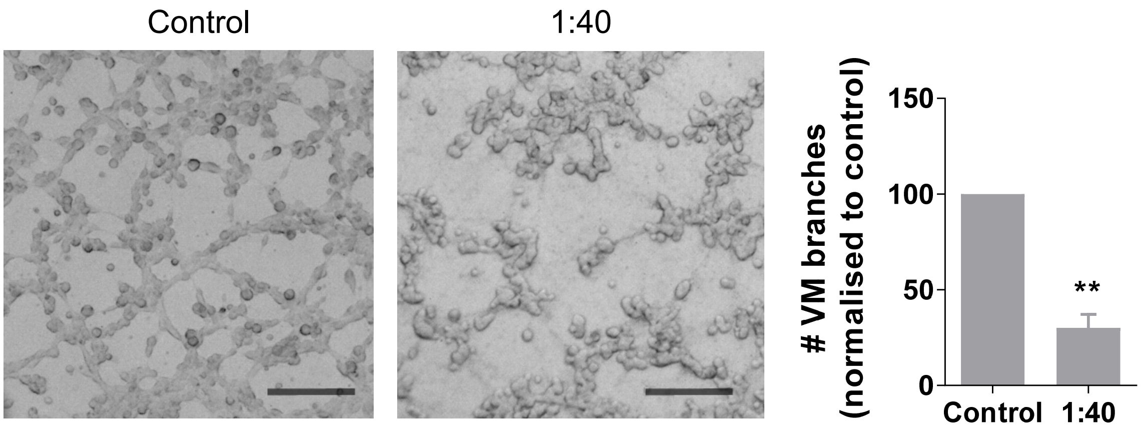

Supplementary Figure 3. Influence of platelets on VM formed structures.

Representative images of C32 melanoma cells treated with platelets 4 hours after VM formation had begun. VM structures are expressed as mean ± SEM for n=3 experiments. ***p*<0.01 compared with buffer control, one-way ANOVA. Scale bar is 200µm, original magnification 40x.

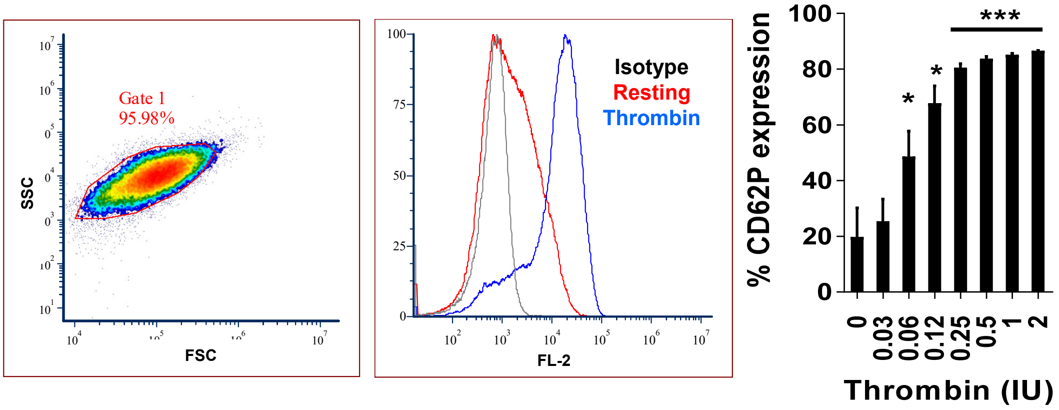

Supplementary Figure 4. Thrombin activation of washed platelets

Platelets purified from healthy human donors were assessed for activation following incubation with human α-thrombin at the indicated concentrations (international unit; IU) for 5 min at 37°C using flow cytometry. Representative flow cytometry scatter plot showing the gated platelet population by forward scatter and side scatter. The population in Gate 1 was stained for expression of CD62P. Curves represent (red) untreated platelets, (grey) isotype control, and (blue) platelets treated with 0.5 IU α-thrombin. Percent expression of platelets stained positive for CD62P. Data are expressed as mean ± SEM. Results are pooled from 3 separate experiments using different platelet donors. *p<0.05, ***p<0.001 vs no treatment control, one-way ANOVA with Bonferroni's post-test.

Supplementary Videos 1-3. Live imaging of platelet influence on VM formation by C32 melanoma cells.

Representative videos of C32 melanoma cells co-cultured without and with platelets at increasing ratios to cancer cells (i.e. cancer cell:platelet of 1:5 and 1:20) in the VM assay over 8 hours confocal live imaging using the CV100 Disk Confocal Microscope.

Supplementary Table 1.

Primer lists for human genes used, including housekeeping genes.

Target gene name	Primer sequence
Human CDH5	Fwd: TGACAATGTCCAAACCCACTCA<br>Rev: TGACAACAGCGAGGTGTAAAGAC
Human KDR	Fwd: ATCACACAATTAAAGCGGGG<br>Rev: CTGGGGTGGGACATACA
Human EPHA2	Fwd: AGACGCTGAAAGCCGGCTAC<br>Rev: CAGGGCCCCATTCTCCATG
Human LAMC2	Fwd: AACCCACAACCCACAACCTT<br>Rev: GCCGAAAGTTATCTGGGCCT
Human MMP1	Fwd: CTGGCCACAACTGCCAAATG<br>Rev: CTGTCCCTGAACAGCCCAGTACTTA
Human MMP2	Fwd: TTTCCATTCCGCTTCCAGGGCAC<br>Rev: TCGCACACCACATCTTTCCGTCACT
Human MMP9	Fwd: CTATTTCTGCCAGGACCGCT<br>Rev: GTTGGTCCCAGTGGGGATTT
Human MMP14	Fwd: GGAGAATTTTGTGCTGCCCG<br>Rev: TTGGTTATTCCTCACCCGCC
**Housekeeping genes**	
Human GAPDH	Fwd: CCATGTTGCAACCGGGAAG<br>Rev: CATCACCCGGAGGAGAAATCG
Human ACTB	Fwd: ATGTACGTTGCTATC<br>Rev: CTCCTTAATGTCACG
Human CYCA	Fwd: GGCAAATGCTGGACCCAACACAAA<br>Rev: CTAGGCATGGGAGGGAACAAGGAA
